# Giant Central Nervous System Aspergilloma Mimicking Butterfly Neoplasm of the Corpus Callosum

**DOI:** 10.7759/cureus.26225

**Published:** 2022-06-23

**Authors:** Kumail Khandwala, Fatima Mubarak, Khurram Minhas, Fatima Gauhar, Anwar Ahmed

**Affiliations:** 1 Radiology, Aga Khan University Hospital, Karachi, PAK; 2 Histopathology, Aga Khan University Hospital, Karachi, PAK; 3 Neurological Surgery, Aga Khan University Medical College, Karachi, PAK

**Keywords:** mass-like lesion, immunocompetent, magnetic resonance, neoplasm, aspergilloma

## Abstract

Radiological presentation of central nervous system (CNS) aspergillosis is variable and depends on the immune status of the patients. Typical features of meningoencephalitis, infarction, abscess, and mycotic aneurysms commonly occur in immunocompromised patients. A rare mass-like or tumoral form of cerebral aspergillosis has been described mostly in immunocompetent patients which results in a diagnostic dilemma, thus potentially causing a delay in the management. We present a case of a large CNS aspergilloma mimicking an infiltrative callosal neoplasm in a young immunocompetent patient. Careful evaluation of imaging features, anatomical location, enhancement pattern, concomitant sinonasal and orbital extension, and angio-aggressive nature of the mass lesion with a high index of suspicion can help diagnose CNS aspergillosis in such patients.

## Introduction

*Aspergillus *species are the most common cause of central nervous system (CNS) fungal infections, with *Aspergillus fumigatus* being the most frequent cause of invasive aspergillosis in immunocompromised hosts and *Aspergillus flavus*, due to greater virulence, the most frequent cause in immunocompetent patients. Clinical manifestations of the disease process depend on the extension of the infection. Symptoms can range from headaches and seizures to focal neurological deficit [1,2].

Radiological presentation of cerebral aspergillosis varies from that of meningoencephalitis, cerebral infarction, cerebral abscess, or mycotic aneurysms, which are findings typically seen in immunocompromised patients. A rare mass-like or tumoral form of CNS aspergillosis has also been described in the literature, mostly encountered in immunocompetent patients, which results in a diagnostic dilemma as the possibility of fungal infection is considered less likely in such patients which can cause a delay in management [3]. We present a case of a large CNS aspergilloma mimicking a neoplasm in a young immunocompetent patient. Through this case report, we attempt to highlight the unusual radiological presentation of this entity and describe the salient imaging features along with appropriate differentials.

## Case presentation

A 26-year-old male, with no known comorbidities, presented to the neurology clinic with complaints of headache and blurring of right-sided vision for the past two months. He had no medical or surgical history. He belonged to the lower socioeconomic stratum, lived on the outskirts of the city, and was a landlord by profession. He had no significant occupational or exposure history except for the presence of cotton factories near his residence. He had no sinonasal symptoms. He was vitally stable with unremarkable general and systemic examinations. His blood workup was normal; the complete blood count showed a total leukocyte count of 8.6 × 10^9^/L (with normal neutrophil and lymphocyte ratios).

**Figure 1:**
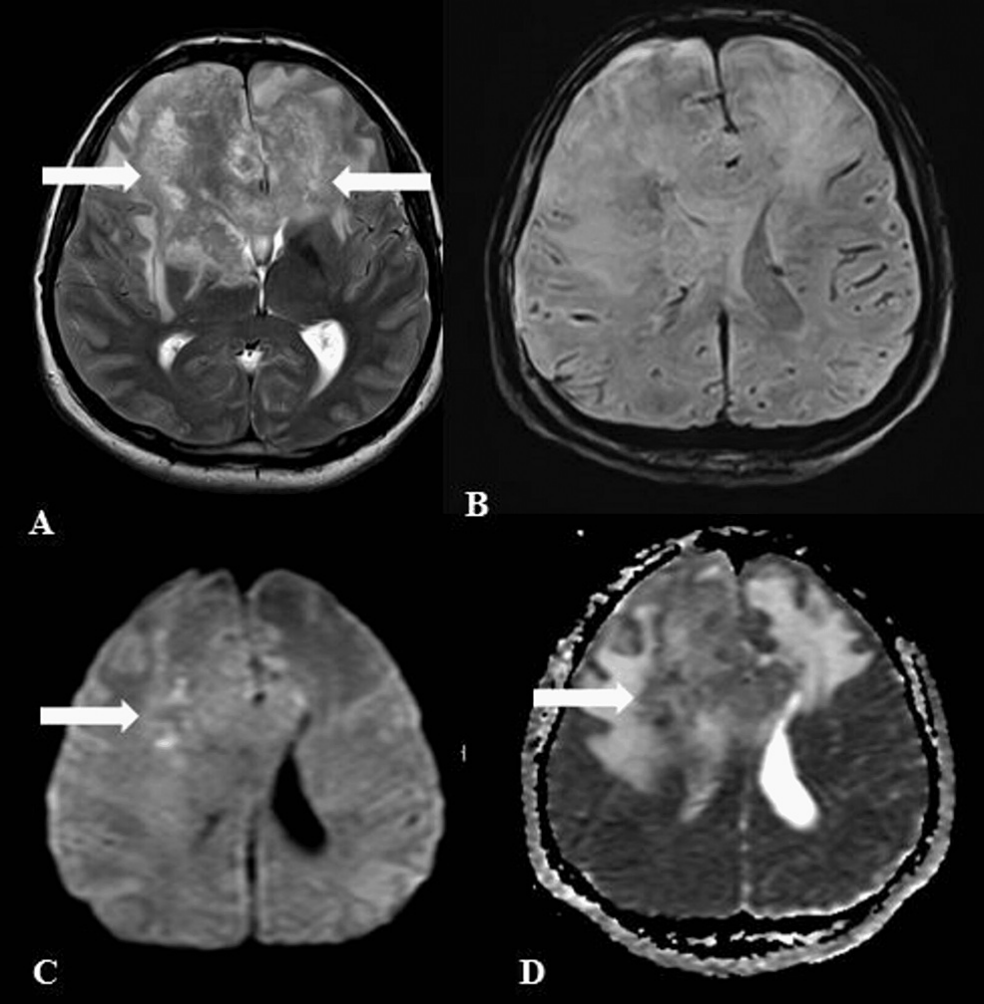
Magnetic resonance images of the brain. Axial T2-weighted sequence (A) showing an infiltrative heterogeneously hypointense lesion in the anterior cranial fossa and frontal lobes, with surrounding perilesional edema (arrows). No susceptibility seen on SWI sequences (B). Patchy foci of diffusion restriction (arrows) noted within it on DWI and ADC maps (C&D). SWI: susceptibility-weighted imaging; DWI: diffusion-weighted imaging; ADC: apparent diffusion coefficient

Magnetic resonance imaging (MRI) performed with a 3-Tesla scanner showed a large infiltrative enhancing lesion in the anterior cranial fossa. It was infiltrating bilateral frontal lobes involving the genu of the corpus callosum and extending into the right-sided basal ganglia. There was an associated mass effect and surrounding edema. The lesion appeared heterogeneously iso to hypointense on T2-weighted sequences and isointense on T1-weighted sequences with avid post-contrast enhancement. There was subtle patchy peripheral diffusion restricted foci within it, with no significant susceptibility on susceptibility-weighted imaging (SWI) (Figure 1).

**Figure 2:**
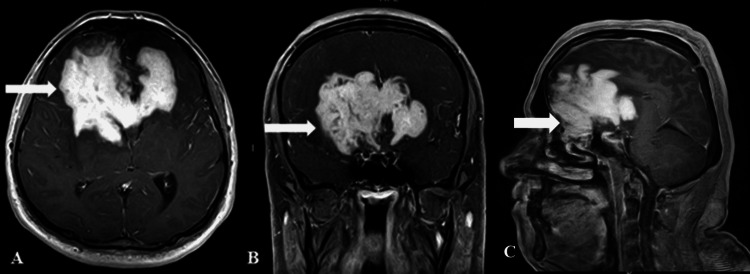
Post-contrast magnetic resonance images of the brain. Axial (A) and coronal (B) post-gadolinium T1-weighted sequences showing an avidly enhancing lesion infiltrating the corpus callosum and bilateral frontal lobes (arrows). On the sagittal image (C), the lesion can be seen infiltrating the floor of the anterior cranial fossa with extension into the ethmoid sinuses (arrow).

The margins of mass were slightly lobulated with frond-like projections. The lesion was also seen eroding the floor of the anterior cranial fossa with extension into the right-sided ethmoid sinuses (Figure 2). The differential diagnoses included a large neoplastic lesion of the corpus callosum, such as butterfly glioma or lymphoma, with the other possibility of an aggressive fungal infection. However, the immunocompetent status of the patient posed a diagnostic dilemma.

**Figure 3:**
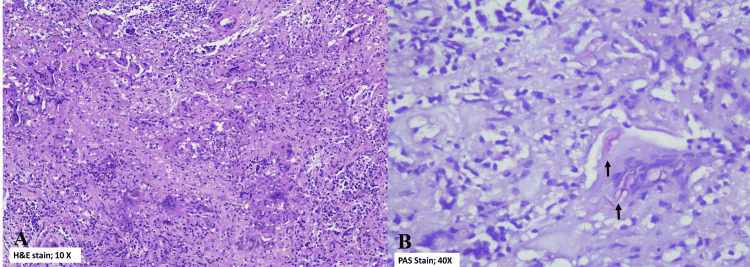
Histology slides. (A) Glial tissue exhibiting foci of chronic granulomatous inflammation and scattered multinucleated giant cells (hematoxylin and eosin stain 10×). (B) Special stain (Periodic acid-Schiff-diastase 40×) highlights septate fungal hyphae (black arrows).

The patient underwent neuro-navigation-guided craniotomy with biopsy and debulking of the frontal space-occupying lesion and right frontal lobectomy under general anesthesia. Intraoperatively, the findings were of a non-hemorrhagic lesion invading the entire right frontal lobe, and this was confirmed to be a fungal infection on the frozen section and histopathology (Figure 3). Tissue fungal culture subsequently grew *Aspergillus flavus* species.

**Figure 4:**
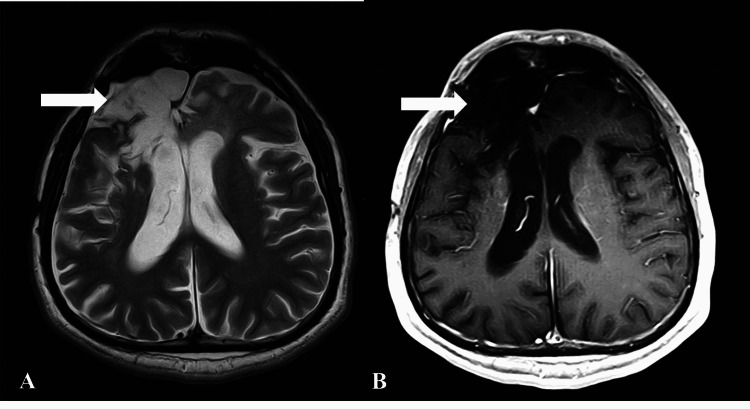
Follow-up magnetic resonance images of the brain. Postoperative and posttreatment scans after one year. (A) Axial T2-weighted sequence shows encephalomalacia at the site of surgery (arrow). (B) No significant residual disease is seen on the axial post-contrast T1-weighted image (arrow).

The patient was discharged on intravenous amphotericin B but came back seven days later due to an episode of unconsciousness lasting for a few minutes and frothing from the mouth. A repeat computed tomography (CT) scan showed residual fungal tissue, and he was treated with intravenous voriconazole. Since then, the patient has presented to the emergency room twice with complaints of progressively increasing seizures of up to four to six episodes per day as of last year and is currently being managed conservatively on lacosamide and levetiracetam. His follow-up imaging after a year of treatment showed significant improvement in the disease process with associated encephalomalacia and gliosis at the site of surgical resection (Figure 4).

## Discussion

The radiological presentation of CNS aspergillosis is variable and depends upon the immune status of the patient. The characteristic findings described in immunodeficient patients can range from ring-enhancing lesions, abscesses, meningitis/meningoencephalitis, and small ischemic or hemorrhagic infarcts due to vasculitis and mycotic aneurysms [4]. In immunocompetent patients, a mass-like or tumoral form of aspergilloma has been described which usually presents as an intracerebral, intracranial-extradural lesion or invading the orbit and/or skull base [3-7].

The diagnostic hint for CNS aspergillosis in our case was the hypointensity in the lesion on T2-weighted sequences on MRI and infiltration into the ethmoid sinuses suggestive of an aggressive process with paranasal sinus involvement. The T2 hypointensity correlates with the presence of paramagnetic elements such as iron, manganese, magnesium, and zinc that are essential for the hyphal growth of the fungi [3]. Strikingly, there was no susceptibility noted in our case on SWI sequences, which have also been characteristically described in fungal infections.

With regards to the differentials of neoplasms of the anterior cranial fossa, lymphoma and meningioma have been described as mimickers of CNS aspergillosis [3,8,9]. Very rarely, a giant aspergilloma may infiltrate the corpus callosum and mimic a butterfly glioma, as stated in one study in our extensive literature search [10]. However, butterfly gliomas have infiltrative margins with areas of necrosis and are usually T2 hyperintense. The lesion in our case mimicked butterfly glioma or lymphoma due to its involvement of the corpus callosum and avid enhancement pattern. Furthermore, the immunocompetent status of the patient posed a diagnostic dilemma and made fungal infection a less likely clinical diagnosis. Callosal involvement by CNS aspergilloma is rare but has been described; however, the symmetrical butterfly appearance in our case makes it unique [8,10].

CNS lymphoma can also involve the paranasal sinuses with coexisting intracranial components, which may be difficult to differentiate from aggressive fungal infection. Lymphomatous lesions usually appear hyperdense on CT and may also be hypointense on T2-weighted sequences on MRI due to high cellular pattern. They exhibit homogenous and avid post-contrast enhancement similar to aspergilloma; however, the involvement of paranasal sinuses, encasement and involvement of the internal carotid arteries, and adjacent calvarial involvement are not frequently encountered. MR spectroscopy demonstrates elevated lipid/lactate peaks and high choline/creatine ratios, and diffusion-weighted imaging shows restriction, which can be differentiating features from CNS aspergilloma [3]. In our case, the lesion also showed avid enhancement and patchy specks of diffusion restriction which made lymphoma a differential; however, a limitation in our report is that MR spectroscopy was not carried out preoperatively which could have helped narrow down the radiological diagnosis.

In contrast to aspergilloma, meningioma appears isointense to gray matter on both T1- and T2-weighted sequences. Both lesions enhance homogeneously on post-contrast sequences. Rarely, meningioma can also arise in the orbit and paranasal sinuses [11]. Fibrous and psammomatous types of meningioma can have T2 hypointense signals similar to aspergilloma; however, these categories of meningioma do not show homogenous, avid contrast enhancement, and adjacent paranasal sinus involvement is also not usually seen [3]. However, malignant transformation of meningioma can simulate an infiltrative lesion like aspergilloma.

In the past, CNS aspergillosis was generally considered a fatal disease. Medical advances have reduced the mortality rate but early diagnosis is crucial. Culture and histopathology remain the gold standard of diagnosis by obtaining infected tissue. Molecular methods and biochemical laboratory analysis such as (1,3)-beta-d-glucan and galactomannan levels can also be used for diagnosis [2]. Treatment of CNS aspergillosis includes surgical excision followed by the use of voriconazole (oral or intravenous) as first-line treatment replacing amphotericin B due to poor CNS penetration and renal and infusion toxicities [1].

## Conclusions

Fungal infections such as cerebral aspergillosis can occur in immunocompetent individuals with no prior history and may present as an intra-axial mass-like lesion and involve the corpus callosum, therefore mimicking a cerebral neoplasm. This form of presentation usually poses a diagnostic dilemma as the possibility of fungal infection is considered less likely in such patients causing a delay in the management. Careful evaluation of MRI features such as signal intensity on different sequences, anatomical location, enhancement pattern, concomitant sinonasal and orbital extension, and angio-aggressive nature of the mass can help diagnose CNS aspergillosis in such patients.
